# Coronal knee alignment is directly related to knee medial‐to‐lateral bone density ratio

**DOI:** 10.1002/jeo2.70719

**Published:** 2026-04-14

**Authors:** Craig E. Klinger, Robert E. Bilodeau, Maximilian M. Mueller, Peter K. Sculco, Scott A. Rodeo, Elizabeth B. Gausden, Hollis G. Potter, Emily M. Stein, Kathryn A. Barth, William M. Ricci, Derek G. Hansen

**Affiliations:** ^1^ Department of Orthopaedic Surgery Hospital for Special Surgery New York New York USA; ^2^ Department of Orthopaedic Surgery Weill Cornell Medicine New York New York USA; ^3^ University of Pittsburgh School of Medicine University of Pittsburgh Pittsburgh Pennsylvania USA; ^4^ Department of Trauma Surgery, Orthopaedics and Sports Traumatology BG Klinikum Hamburg Hamburg Germany

**Keywords:** bone density, distal femur, hip–knee–ankle angle, medial‐to‐lateral ratio, proximal tibia

## Abstract

**Purpose:**

To assess associations between coronal knee alignment and regional epiphyseal bone mineral density (BMD) using computed tomography (CT)‐derived Hounsfield units (HU) and compare medial‐to‐lateral ratios (MLRs) across alignment groups, and as a secondary aim, to evaluate the association of sex and demographic factors with regional BMD patterns.

**Methods:**

A retrospective search (2008–2025) identified patients with non‐contrast knee CT and standing anteroposterior long‐leg radiographs. Exclusions included prior femur or tibia fracture or surgery and metabolic bone disease other than osteopenia or osteoporosis. Mean trabecular HU were measured for medial and lateral femoral condyles (MFC, LFC), distal femur epiphysis (DFE), medial and lateral tibial plateaus (MTP, LTP) and proximal tibia epiphysis (PTE). Outcomes included DFE‐MLR (MFC/LFC), PTE‐MLR (MTP/LTP) and combined‐MLR ([MFC + MTP]/[LFC + LTP]). Alignment was categorized by hip–knee–ankle angle (HKAA) as major varus, varus, neutral, valgus or major valgus.

**Results:**

The cohort included 312 patients (mean age 66.5 ± 9.3 years; 50.6% female); 99.4% underwent CT for robotic‐assisted arthroplasty planning. LFC HU exceeded MFC HU in 88.5% of cases (*p* < 0.001), and MTP HU exceeded LTP HU in 92.9% (*p* < 0.001). Increasing valgus correlated with decreasing MLR (DFE‐MLR *r* = −0.459; PTE‐MLR *r* = −0.322; combined‐MLR *r* = −0.509; all *p* < 0.001). Females demonstrated higher PTE‐MLR and combined‐MLR than males (*p* ≤ 0.049).

**Conclusions:**

Coronal knee alignment was strongly associated with compartmental epiphyseal BMD MLR, with lower MLR in valgus alignment and greater alignment sensitivity in females. These findings provide quantitative imaging context relevant to arthroplasty planning.

**Level of Evidence:**

Level III, retrospective comparative study.

## Graphical abstract

Coronal knee alignment is associated with medial–lateral differences in trabecular bone density, with increasing valgus alignment linked to lower medial‐to‐lateral bone density ratios.

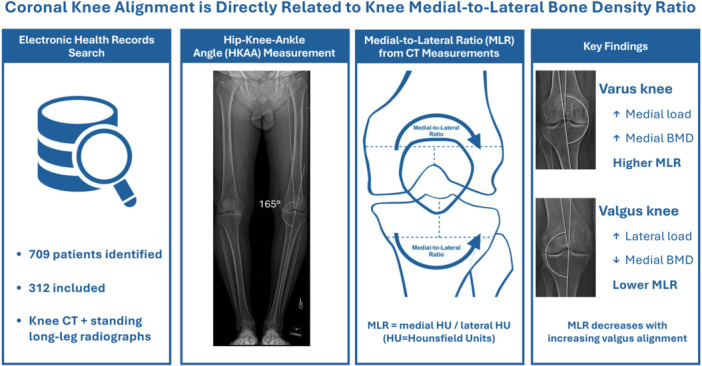

**Abbreviations**
AP: anteroposterior
BMD: bone mineral density
BMI: body mass index
CT: computed tomography
DFE: distal femur epiphysis
DXA: dual‐energy x‐ray absorptiometry
EHR: electronic health record
HKAA: hip–knee–ankle angle
HU: Hounsfield units
ICC: intraclass correlation coefficient
IQR: interquartile range
LFC: lateral femoral condyle
LLXR: long‐leg x‐rays
LTP: lateral tibial plateau
MFC: medial femoral condyle
MLR: medial‐to‐lateral ratio
MTP: medial tibial plateau
OA: osteoarthritis
PTE: proximal tibia epiphysis
TKA: total knee arthroplasty
UKA: unicompartmental knee arthroplasty

## INTRODUCTION

Distal femur and proximal tibia bone density impact periprosthetic fracture risk and outcomes following knee arthroplasty [1, 2, 18]. A study by Kang et al. found that a central dual‐energy x‐ray absorptiometry (DXA) *T* score below −2.8 was a significant independent risk factor for perioperative distal femur fracture during total knee arthroplasty (TKA) [7]. Andersen et al. demonstrated that low preoperative bone mineral density (BMD) is associated with increased tibial component migration in uncemented TKA [1]. Decreased BMD has been found to increase stress fracture risk after unicompartmental knee arthroplasty (UKA) [20]. A review by Deans et al. reported that uncemented TKA in patients with decreased BMD can expect migration within the first 6–12 months, with subsequent increased risk of failed osseous integration [5].

Bone adapts to mechanical loading, with mass and strength increasing in regions of higher habitual stress and decreasing where loads are reduced, as described by Wolff's law [15]. Mechanostat theory further proposes that osteocytes detect local mechanical strain and regulate bone remodelling, promoting bone formation in more heavily loaded regions [17]. These principles provide a biological basis for compartment‐specific differences in knee BMD with altered coronal alignment.

Prior studies have demonstrated an association between knee alignment and compartmental BMD. Rougereau et al. reported systematic variation in distal femoral medial‐to‐lateral BMD ratio (MLR) with hip–knee–ankle angle (HKAA) using computed tomography (CT)‐derived Hounsfield units (HU), with higher MLR in varus knees and lower MLR in valgus deformities [12]. However, analysis was limited to single‐slice femoral regions, and proximal tibial BMD was not assessed. Yoon et al. similarly demonstrated higher medial and lower lateral femoral and tibial BMD with increasing varus alignment using compartment‐specific DXA [21]. DXA‐based methods are projectional and cannot isolate trabecular versus cortical bone, limiting detailed regional assessment.

Accordingly, CT‐based characterization of medial‐to‐lateral epiphyseal BMD for both the distal femur and proximal tibia, and potential sex‐based differences, remains incompletely defined. The primary aim of this study was to assess associations between coronal knee alignment and regional epiphyseal BMD using CT‐derived HU and compare MLR across alignment groups. The secondary aim was to evaluate the association of sex and demographic factors with regional BMD patterns. The hypothesis was that increasing coronal deformity would be associated with higher BMD in the mechanically loaded compartment.

## MATERIALS AND METHODS

An electronic health records (EHRs) search (Epic; Epic Systems) was performed at a single academic institution from 1 January 2008 to 16 August 2025 to identify patients with phantomless, non‐contrast CT, including the knee and standing anteroposterior (AP) long‐leg x‐rays (LLXR). The study institution is an academic tertiary referral hospital that specializes in providing musculoskeletal care.

Patients with a history of femoral or tibial fracture, prior knee surgery (including arthroscopy, ligament reconstruction or arthroplasty), incomplete knee CT coverage, CT acquisition at energies other than 120 kV, an interval greater than 12 months between CT and LLXR, or known metabolic bone disease other than osteopenia or osteoporosis were excluded. Patients with prior fragility fractures at sites other than the femur or tibia and those undergoing medical treatment for osteoporosis were not excluded, as the primary objective was to assess regional medial‐to‐lateral bone density patterns in a representative arthroplasty population. To minimize sampling bias within the study cohort, all patients who met eligibility criteria during the study period were included in this consecutive series.

### Outcome measures

The primary outcome measures were regional MLR (DFE‐MLR: MFC/LFC, PTE‐MLR: MTP/LTP, combined‐MLR: MFC + MTP/LFC + LTP) assessed using CT‐HU measurements and extent of coronal deformity defined by HKAA. Secondary outcome measures assessed the association between additional independent variables, including age, sex and other demographic data, on regional BMD.

### Radiographic measurements and data collection

The HU Measurement protocol was predefined (Supporting Information S1: Data 1). Mean trabecular bone CT‐HU were measured on multiplanar reconstruction using three consecutive axial images (5‐mm slab thickness and step size) on PACS (Sectra IDS7; Sectra). HU measurements excluded the subchondral plate, visible sclerotic bone margins and any bone cysts. Regions of interest included the medial femoral condyle (MFC), lateral femoral condyles (LFC), distal femur epiphysis (DFE), medial tibial plateau (MTP), lateral tibial plateaus (LTP) and proximal tibial epiphysis (PTE). Measurements excluded the subchondral plate, visible sclerosis and cysts. For distal femur measurements, the coronal plane was adjusted to align with the distal medial and LFC margins, and the sagittal plane to align with the distal femur axis, including the metaphysis and epiphysis. HU measurements began at the postero‐superior apex of the femoral intercondylar notch and progressed distally on three consecutive images. For proximal tibia measurements, the coronal plane was adjusted to align with the distal margin of the medial and LTP subchondral plate, and the sagittal plane with the tibial axis.

Coronal knee alignment was measured using the mechanical axis on full‐weight‐bearing AP long‐leg radiographs. HKAA was defined as the angle between the mechanical axes of the femur and tibia, as described by Gielis et al. [6]. All HU and HKAA measurements were performed by the first author.

**Figure 1:**
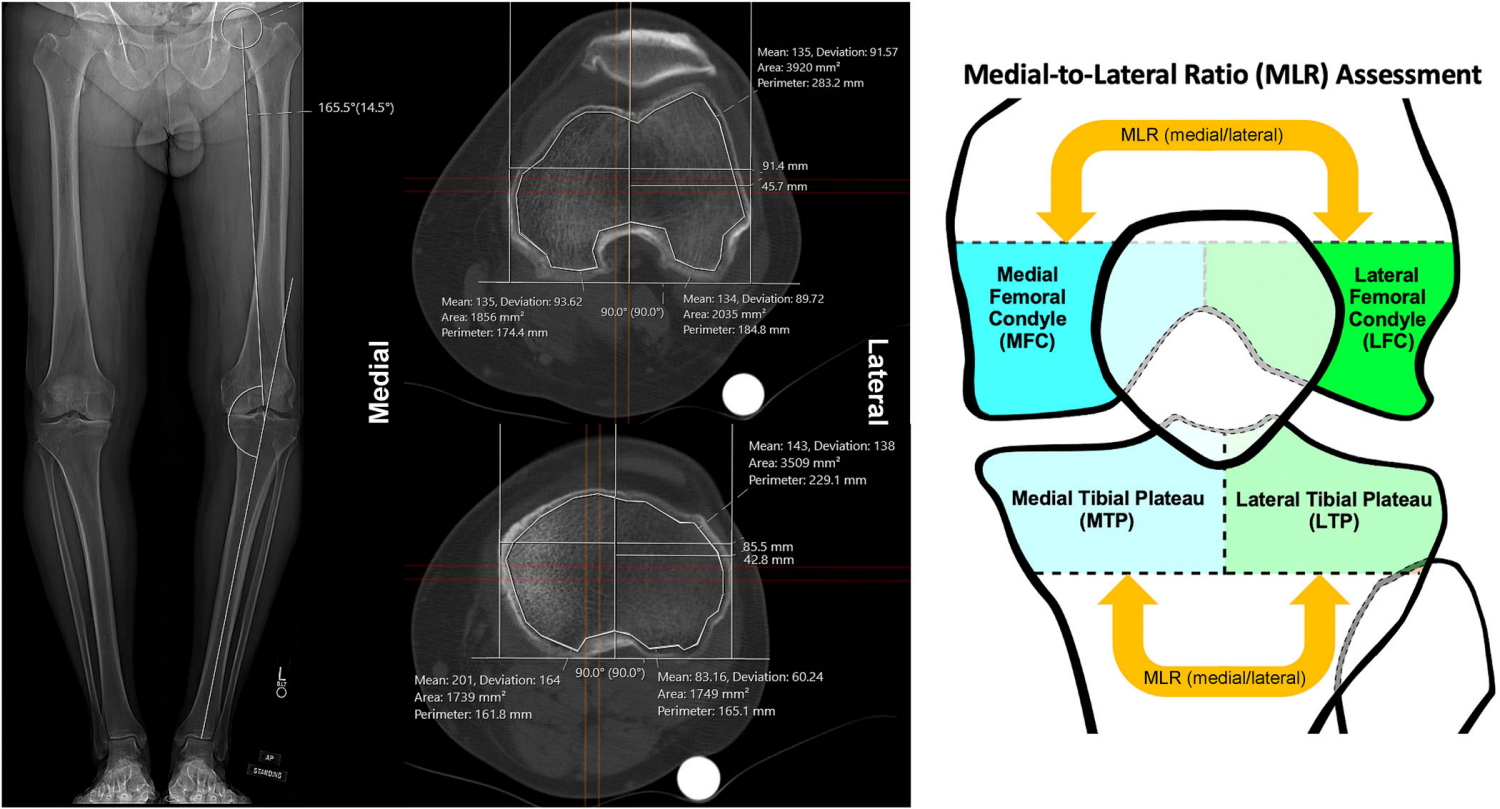
Image illustrating HKAA measurements on a left knee on standing AP LLXR of a major varus case (left image), example HU measurements of the same case including distal femur epiphysis, and proximal tibia epiphysis, and medial and lateral compartments (centre images), and diagram illustrating calculation of the medial‐to‐lateral HU ratio (MLR) for trabecular bone density comparison (right image). AP, anteroposterior; HKAA, hip–knee–ankle angle; HU, Hounsfield units; LLXR, long‐leg x‐rays; MLR, medial‐to‐lateral ratio.

MLR was calculated using regional mean CT‐HU values (DFE: MFC/LFC, PTE: MTP/LTP) (Figure 1). Combined‐MLR was calculated with summed mean regional HU values (MFC + MTP/LFC + LTP) to assess overall compartment MLR. This composite ratio was intended to summarize overall medial‐versus‐lateral trabecular bone density across the knee joint for a global assessment of compartmental adaptation to coronal alignment. MLR and combined‐MLR were assessed by alignment groups defined by Rougereau et al. [12]: major varus (≤170°), varus (>170°, <178°), neutral (178°–182°), valgus (>182°, <190°) and major valgus (≥190°). Clinicodemographic data were collected using EHR. Clinicodemographic variables collected included age, sex, race and ethnicity, height, weight, body mass index (BMI), CT indication and laterality, time between CT and radiographs, osteoporosis status and osteoporosis medication use.

### Statistical analysis

Statistical analysis was performed using SPSS (version 29.0.2.0; IBM). Data were tested for normal distribution with the Shapiro–Wilk test. Correlation coefficients were calculated to assess associations between variables and were interpreted for the strength of association [13]. Data are presented as mean and standard deviation when normally distributed and median and interquartile range (IQR) for nonparametric data (IQR: 25%–75%). An independent samples *T* test or analysis of variance (ANOVA) test was used to analyse normally distributed continuous variables and a Mann–Whitney *U* test or Kruskal–Wallis test for nonparametric data. A *p* value of <0.05 was considered statistically significant. Multivariable linear regression analyses were performed to evaluate whether coronal alignment (HKAA) was independently associated with medial‐to‐lateral bone density ratios after adjusting for potential confounders, including age, sex and BMI. Regression coefficients with 95% confidence intervals were reported. Multicollinearity was assessed using variance inflation factors. Assessment of HU measurement intra‐rater and inter‐rater reliability was performed using intraclass correlation coefficients (ICCs). For intra‐rater reliability, HU measurements were repeated by the first author on a random sample of 30 study patients, three months after initial HU measurements. A second investigator (second author) separately measured HU on the random sample of 30 study patients to assess inter‐rater reliability.

### Ethical aspects

This retrospective study was approved by our Institutional Review Board. The requirement for written informed consent was waived due to the retrospective design and use of de‐identified data. All procedures were conducted in accordance with institutional guidelines and the principles of the Declaration of Helsinki.

## RESULTS

### Descriptive data

**Figure 2:**
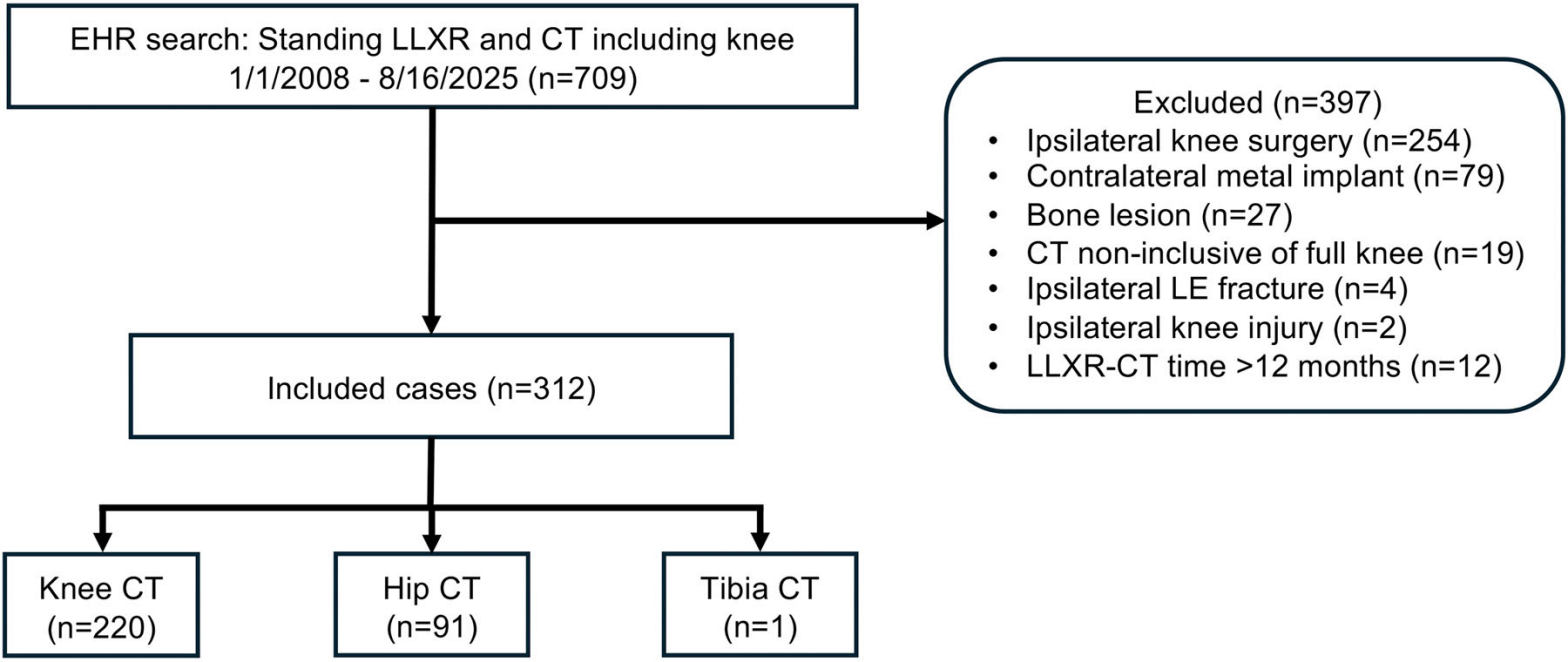
Flowchart of the screening process based on study inclusion and exclusion criteria. BMD, bone mineral density; CT, computed tomography; EHR, electronic health record; LE, lower extremity; LLXR, long‐leg x‐ray.

The EHR search identified 709 patients, of whom 312 met eligibility criteria and were included (mean age 66.5 ± 9.1 years; 158 females, 154 males; Figure 2, Table 1). Age did not differ by sex (67.1 ± 8.9 vs. 65.9 ± 9.6 years; *p* = 0.237). Most patients underwent CT for robotic‐assisted arthroplasty planning (knee 70.5%, hip 28.8%), and 11.2% had documented osteoporosis treatment.

**Table 1:** Baseline characteristics and radiographic details.

Variables (*N* = 312)	Mean (SD)	Range	*N* (%)
Age	66.5 (9.1)	46–91	312 (100.0)
Female (*N* = 158)	67.1 (8.9)	46–88	158 (50.6)
Male (*N* = 154)	65.9 (9.6)	47–91	154 (49.4)
BMI	28.4 (5.3)	16.4–48.0	312 (100.0)
Underweight (<18.5)			2 (0.6)
Normal (18.5–24.9)			84 (26.9)
Overweight (25–29.9)			110 (35.3)
Obese (30–40)			107 (34.3)
Severely obese (>40)			9 (2.9)
Class I Obesity (30–34.9)			84 (26.9)
Class II Obesity (35–39.9)			23 (7.4)
Class III Obesity (40–50)			9 (2.9)
Class IV Obesity (>50)			0 (0.0)
Race			
White			275 (88.1)
Unknown			19 (6.1)
Black			11 (3.5)
Asian			6 (1.9)
American Indian or Alaska Native			1 (0.3)
Ethnicity			
Not Hispanic or Latino			291 (93.3)
Hispanic or Latino			15 (4.8)
Unknown			6 (1.9)
Indication for CT scan			
Lower limb alignment assessment			2 (0.6)
Robotic‐assisted THA, TKA or UKA			310 (99.4)
Unicondylar knee arthroplasty			180 (58.1)
Total hip arthroplasty			90 (29.0)
TKA			39 (12.6)
Patellofemoral arthroplasty			1 (0.3)
Knee used for CT Hounsfield unit measurements			
Right			150 (48.1)
Left			162 (51.9)
Days between standing long‐leg radiographs and CT	15.6 (39.0)	0.0–273.0	312 (100.0)
Undergoing osteoporosis treatment			
Yes			35 (11.2)
No			277 (88.8)

Abbreviations: BMI, body mass index; CT, computed tomography; THA, total hip arthroplasty; SD, standard deviation; TKA, total knee arthroplasty; UKA, unicompartmental knee arthroplasty.

**Figure 3:**
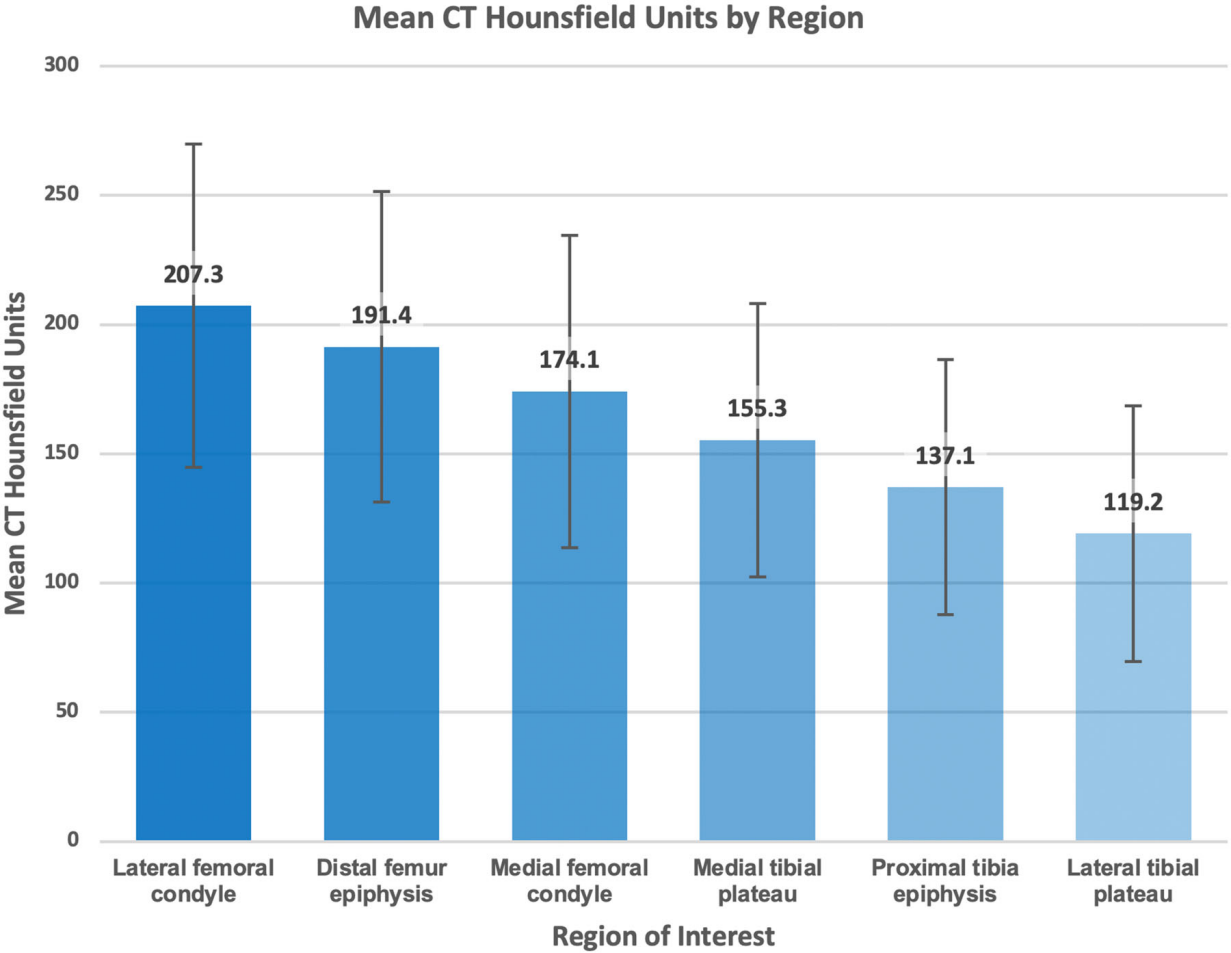
Overall regional mean CT HU (±SD). The lateral femoral condyle had highest BMD based on mean HU, and lateral tibial plateau had the least. The distal femur epiphysis had higher mean BMD than the proximal tibia epiphysis in 99.4% of cases (310/312). BMD, bone mineral density; CT, computed tomography; HU, Hounsfield units; SD, standard deviation.

Across the cohort, mean HU values were highest in the LFC and lowest in the LTP (Figure 3). Higher BMD was observed in the LFC compared with the MFC in 88.5% of cases (*p* < 0.001) and in the MTP compared with the LTP in 92.9% (*p* < 0.001). Increasing valgus alignment was associated with decreasing MLRs at both the DFE (*r* = −0.459, *p* < 0.001) and PTE (*r* = −0.322, *p* < 0.001).

Mean BMD was higher at the DFE than PTE (191.4 vs. 137.1 HU), corresponding to approximately 39.6% higher mean BMD at the distal femur (*p* < 0.001). Within the distal femur, LFC BMD exceeded MFC BMD by approximately 19.1% on average (*p* < 0.001), whereas within the proximal tibia, MTP BMD exceeded LTP BMD by approximately 30.3% (*p* < 0.001). Reversal of these medial–lateral patterns occurred in a minority of cases and varied by alignment group.

### Primary outcome

Most knees demonstrated varus alignment (53.5% varus, 18.3% neutral, 17.0% major varus, 10.6% valgus and 0.6% major valgus; Table 2). HKAA was moderately negatively correlated with distal femoral epiphyseal medial‐to‐lateral ratio (DFE‐MLR; *r* = −0.459, *p* < 0.001) and proximal tibial epiphyseal medial‐to‐lateral ratio (PTE‐MLR; *r* = −0.322, *p* < 0.001), and more strongly correlated with combined‐MLR (*r* = −0.509, *p* < 0.001; Table 3). DFE‐MLR and PTE‐MLR were moderately positively correlated (*r* = 0.320, *p* < 0.001).

**Table 2:** Knee alignment groups based on HKAA.

HKAA group	*N* (%)	Boundaries	Median (IQR)
Study cohort (*n* = 312)			
Overall	312 (100%)	NA	174.7° (171.3–178.5°)
Major varus	53 (17.0%)	≤170°	167.9° (165.9–169.2°)
Varus	167 (53.5%)	>170°, <178°	173.8° (172.1–175.5°)
Neutral	57 (18.3%)	178°–182°	179.4° (178.6–180.5°)
Valgus	33 (10.6%)	>182°, <190°	184.8° (182.7–186.0°)
Major valgus	2 (0.6%)	≥190°	192.3° (191.2–193.3°)
Females (*n* = 158)			
Overall	158 (100%)	NA	176.9° (173.1–180.6°)
Major varus	19 (12.0%)	≤170°	168.1° (166.9–169.7°)
Varus	69 (43.7%)	>170°, <178°	174.5° (172.8–176.3°)
Neutral	43 (27.2%)	178°–182°	179.5° (178.6–180.8°)
Valgus	26 (16.5%)	>182°, <190°	184.5° (182.5–185.8°)
Major valgus	1 (0.6%)	≥190°	190.2° (190.2–190.2°)
Males (*n* = 154)			
Overall	312 (100%)	NA	173.1° (170.6–176.2°)
Major Varus	34 (22.1%)	≤170°	167.3° (165.8–169.1°)
Varus	98 (63.6%)	>170°, <178°	173.5° (172.0–175.0°)
Neutral	14 (9.1%)	178°–182°	179.2° (178.6–179.7°)
Valgus	7 (4.5%)	>182°, <190°	186.0° (185.7–186.9°)
Major valgus	1 (0.6%)	≥190°	194.3° (193.3–194.3°)

Abbreviations: HKAA, hip–knee–ankle angle; IQR, interquartile range (IQR 25%–75%); NA, not applicable.

**Table 3:** Correlations between variables.

Variables	Correlation coefficient^a^	*p* value
HKAA and DFE‐MLR	−0.459	<0.001
HKAA and PTE‐MLR	−0.322	<0.001
DFE‐MLR and PTE‐MLR	0.320	<0.001
Combined‐MLR and HKAA	−0.509	<0.001
HKAA and Bodyweight	−0.331	<0.001
HKAA and Height	−0.198	<0.001
HKAA and BMI	−0.285	<0.001
Age and DFE‐MLR	−0.219	<0.001
Age and PTE‐MLR	0.102	0.072
Age and Combined‐MLR	−0.083	0.288
Age and MFC HU	−0.247	<0.001
Age and LFC HU	−0.160	0.005
Age and MTP HU	−0.210	<0.001
Age and LTP HU	−0.257	<0.001
Bodyweight and MFC HU	0.348	<0.001
Bodyweight and LFC HU	0.345	<0.001
Bodyweight and MTP HU	0.355	<0.001
Bodyweight and LTP HU	0.343	<0.001
Height and MFC HU	0.280	<0.001
Height and LFC HU	0.314	<0.001
Height and MTP HU	0.238	<0.001
Height and LTP HU	0.313	<0.001
BMI and MFC HU	0.234	<0.001
BMI and LFC HU	0.203	<0.001
BMI and MTP HU	0.278	<0.001
BMI and LTP HU	0.203	<0.001

Abbreviations: BMI, body mass index; Combined‐MLR, MFC + MTP to LFC + LTP ratio; DFE, distal femur epiphysis; HKAA, hip–knee–ankle angle; HU, Hounsfield units; LFC, lateral femoral condyle; LTP, lateral tibial plateau; MFC, medial femoral condyle; MLR, medial‐to‐lateral ratio; MTP, medial tibial plateau; PTE, proximal tibia epiphysis.

^a^ Pearson correlation coefficient.

**Figure 4:**
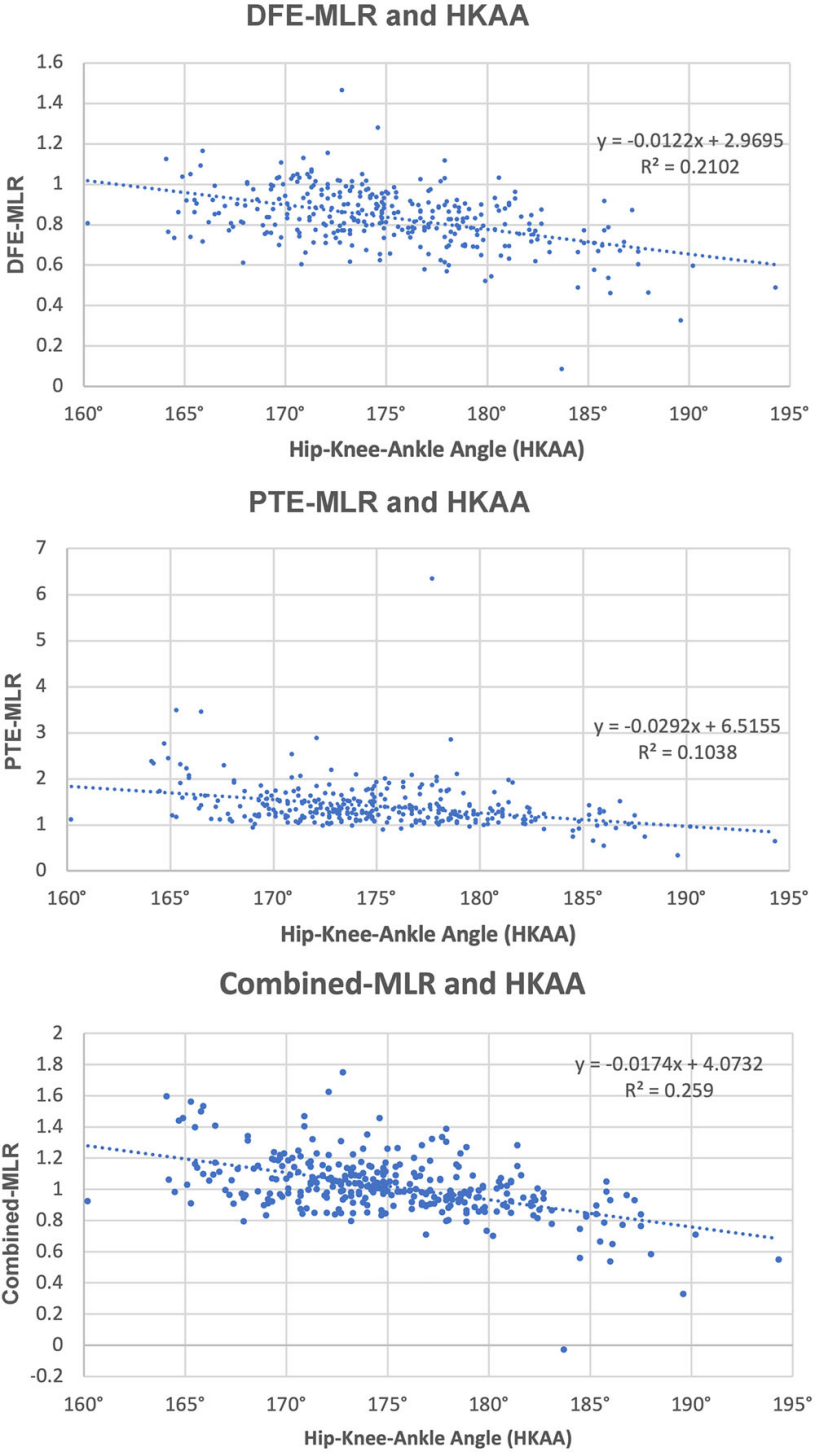
Scatterplot of overall correlations (*n* = 312) between DFE‐MLR and HKAA (top), PTE‐MLR and HKAA (middle) and combined‐MLR and HKAA (bottom). DFE, distal femur epiphysis; HKAA, hip–knee–ankle angle; MLR, medial‐to‐lateral ratio; PTE, proximal tibia epiphysis.

Across increasing varus alignment, a graded increase in medial‐to‐lateral BMD was observed at both the distal femur and proximal tibia, reflected by progressively higher DFE‐MLR, PTE‐MLR and combined‐MLR values (Table 4, Figure 4). Significant differences in MLR were observed between alignment groups for DFE, PTE and combined‐MLR (Supporting Information S2: Table 1). In neutral alignment, MFC BMD was 20.4% lower than LFC BMD (*p* = 0.002), and LTP BMD was 23.5% lower than MTP BMD (*p* = 0.002).

**Table 4:** MLR BMD measurements by knee alignment group.

HKAA group	*N* (%)	Median (IQR)
Distal femur epiphysis MLR (*N* = 312)		
Overall	312 (100)	0.83 (0.76–0.93)
Major varus	53 (17.0)	0.88 (0.80–0.99)
Varus	167 (53.5)	0.87 (0.78–0.96)
Neutral	57 (18.3)	0.80 (0.70–0.88)
Valgus	33 (10.6)	0.71 (0.62–0.77)
Major valgus	2 (0.6)	0.54 (0.51–0.57)
Proximal tibia epiphysis MLR (*N* = 312)		
Overall	312 (100)	1.29 (1.13–1.55)
Major varus	53 (17.0)	1.45 (1.21–1.90)
Varus	167 (53.5)	1.33 (1.17–1.56)
Neutral	57 (18.3)	1.24 (1.14–1.41)
Valgus	33 (10.6)	1.07 (0.92–1.24)
Major valgus	2 (0.6)	0.80 (0.72–0.88)
Combined‐MLR (*N* = 312)		
Overall	312 (100)	1.00 (0.92–1.10)
Major varus	53 (17.0)	1.09 (0.97–1.20)
Varus	167 (53.5)	1.04 (0.96–1.12)
Neutral	57 (18.3)	0.96 (0.89–1.02)
Valgus	33 (10.6)	0.84 (0.76–0.93)
Major valgus	2 (0.6)	0.63 (0.59–0.67)

Abbreviations: BMD, bone mineral density; Combined‐MLR, MFC + MTP to LFC + LTP ratio; IQR, interquartile range; MLR, medial‐to‐lateral ratio.

### Secondary outcomes

Moderate positive correlations were found between bodyweight and HU for all medial and lateral regions (MFC: *r* = 0.280, LFC: *r* = 0.314, MTP: *r* = 0.238, LTP: *r* = 0.313; *p* < 0.001) (Table 3). Moderate positive correlations were also found between BMI and HU for all medial and lateral regions (MFC: *r* = 0.234, LFC: *r* = 0.203, MTP: *r* = 0.278, LTP: *r* = 0.203; *p* < 0.001). Moderate negative correlations were found between age and HU for MFC, MTP and LTP (*r* = −0.247; *r* = −0.210, *r* = −0.257, respectively; all *p* < 0.001) and weak negative correlations for age and HU for LFC (*r* = −0.160, *p* = 0.005).

### Stratified analysis by sex

A stratified analysis was performed to evaluate sex‐based differences. Median HKAA was greater in females than males (176.9° [173.1°–180.6°] vs. 173.1° [170.6°–176.2°], *p* < 0.001), indicating more varus alignment among males (Supporting Information S2: Table 2). Alignment group distributions differed by sex, with males demonstrating a higher prevalence of varus and major varus deformity.

**Figure 5:**
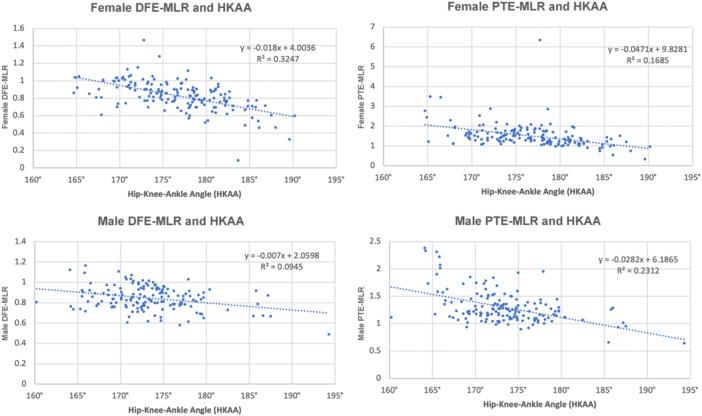
Scatterplot of correlations stratified by sex between DFE‐MLR and HKAA, and correlation between PTE‐MLR and HKAA, with correlations for females on top (*n* = 158) and for males on bottom (*n* = 154). A stronger association was observed in females compared with males, particularly for the distal femur. DFE, distal femur epiphysis; HKAA, hip–knee–ankle angle; MLR, medial‐to‐lateral ratio; PTE, proximal tibia epiphysis.

**Figure 6:**
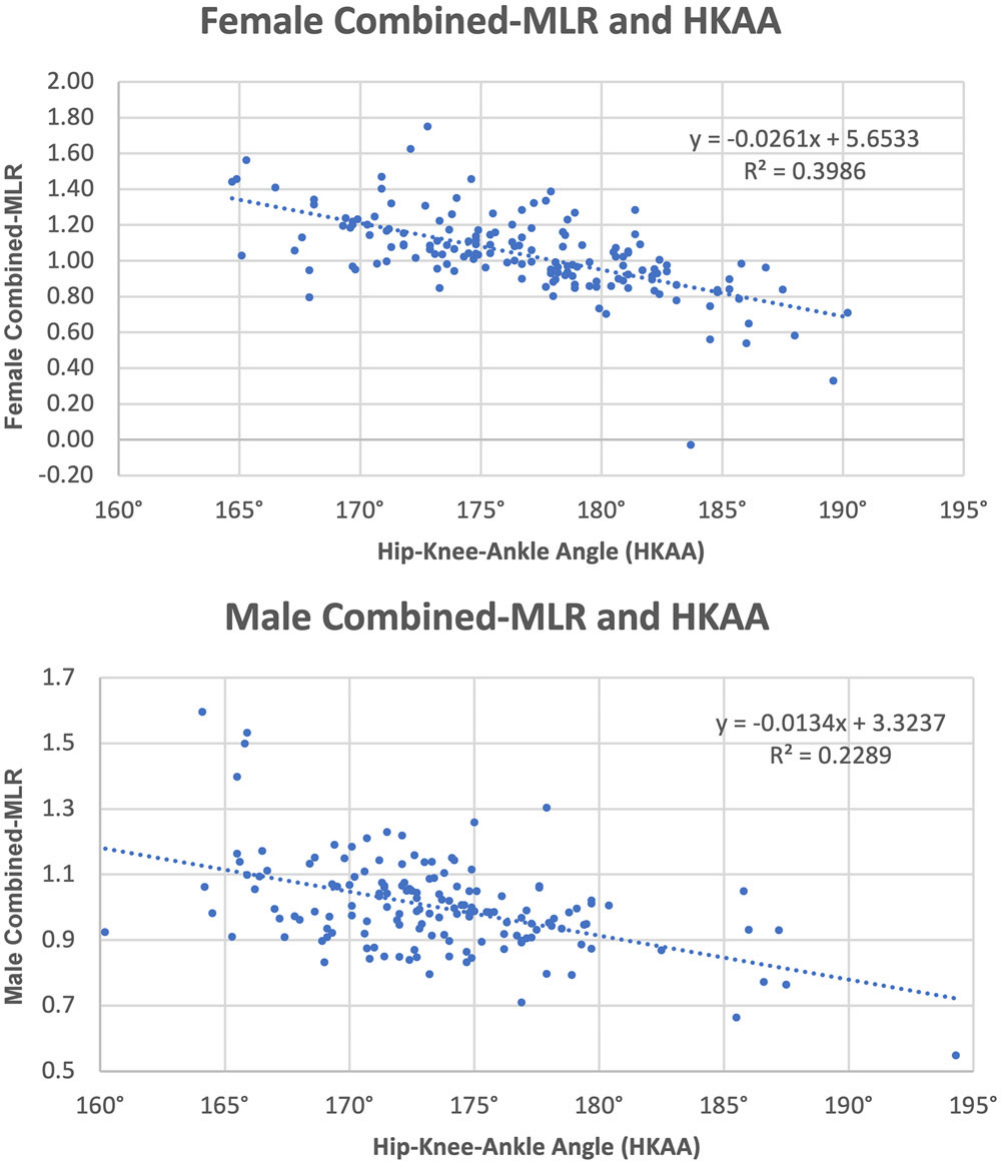
Scatterplot of correlations stratified by sex between Combined‐MLR (MFC + MTP divided by LFC + LTP) and HKAA including for females (top; *n* = 158) and males (bottom; *n* = 154), with a stronger association observed in females. Combined‐MLR, MFC + MTP to LFC + LTP ratio; HKAA, hip–knee–ankle angle; LFC, lateral femoral condyle; LTP, lateral tibial plateau; MFC, medial femoral condyle; MLR, medial‐to‐lateral ratio; MTP, medial tibial plateau.

DFE‐MLR did not differ by sex (males 0.84 vs. females 0.83, *p* = 0.407). In contrast, proximal tibial epiphyseal MLR (PTE‐MLR) and combined‐MLR were significantly higher in females than males (PTE‐MLR: 1.39 vs. 1.22, *p* < 0.001; combined‐MLR: 1.03 vs. 0.99, *p* = 0.049). In both sexes, increasing varus alignment was associated with progressively higher medial epiphyseal BMD at the distal femur and proximal tibia, reflected by increasing DFE‐MLR, PTE‐MLR and combined‐MLR values (Supporting Information S2: Table 2; Figures 5, 6 and 6).

Sex‐specific differences were observed in the strength of alignment–MLR associations. The negative correlation between HKAA and DFE‐MLR was stronger in females than males, whereas the negative correlation between HKAA and PTE‐MLR was stronger in males (Supporting Information S2: Table 3). Combined‐MLR demonstrated a strong negative correlation with HKAA in both females and males.

More alignment‐group differences were observed among females than males for DFE‐MLR, PTE‐MLR and combined‐MLR (Supporting Information S2: Table 4). In contrast, only limited between‐group differences were identified among males, primarily between valgus and major varus alignment groups.

### Multivariable linear regression

In multivariable linear regression models adjusting for age, sex and BMI, coronal alignment demonstrated a consistent association with medial‐to‐lateral bone density ratios across all regions. For DFE‐MLR, increasing valgus alignment (higher HKAA) was associated with lower MLR (*β* = −0.013 per degree; 95% confidence interval [CI]: −0.016 to −0.010; *p* < 0.001), with the model explaining 27.3% of the variance (adjusted *R* ^2^ = 0.264). Older age was independently associated with lower DFE‐MLR (*β* = −0.004 per year; *p* < 0.001), while female sex was associated with slightly higher DFE‐MLR (*β* = 0.031; *p* = 0.040). BMI was not independently associated with DFE‐MLR.

For PTE‐MLR, HKAA again demonstrated a strong inverse association (*β* = −0.036 per degree; 95% CI −0.046 to −0.026; *p* < 0.001). Female sex was the strongest independent predictor of higher PTE‐MLR (*β* = 0.334; *p* < 0.001), whereas age and BMI were not independently associated with PTE‐MLR. This model accounted for 21.6% of the variance (adjusted *R* ^2^ = 0.206).

The combined‐MLR demonstrated the strongest overall association with alignment, with higher HKAA independently associated with lower combined‐MLR (*β* = −0.020 per degree; 95% CI −0.024 to −0.017; *p* < 0.001). Female sex remained independently associated with higher combined‐MLR (*β* = 0.104; *p* < 0.001), while age and BMI were not significant predictors. The combined‐MLR model explained 33.1% of the variance (adjusted *R* ^2^ = 0.323). No evidence of multicollinearity was observed in any model (all variance inflation factors <1.2).

### Reliability analysis

HU measurements for all regions demonstrated excellent intra‐rater reliability (range: 0.971–0.999) and excellent inter‐rater reliability (range: 0.968–0.999).

## DISCUSSION

A primary study finding was that increasing varus alignment was associated with progressively greater medial BMD at both the distal femur and proximal tibia. This relationship was strongest when MLRs were assessed across combined epiphyseal regions and was more pronounced in females than in males. The stronger association observed with combined‐MLR likely reflects the fact that coronal mechanical loading across the knee is transmitted through both the femoral condyle and corresponding tibial plateau within a given compartment. Summation of medial femoral and medial tibial trabecular attenuation relative to the lateral compartment, therefore, represents a composite marker of cumulative compartmental adaptation along the primary load‐bearing pathway. Because alignment‐related load redistribution affects the entire articular column rather than an isolated surface, the combined metric may better capture global coronal load effects than region‐specific ratios alone. Females demonstrated stronger alignment–MLR correlations and greater between‐group differences, suggesting sex‐specific sensitivity of regional bone adaptation to coronal deformity.

Higher proximal tibial medial‐to‐lateral BMD ratios observed in females may reflect sex‐specific differences in bone remodelling sensitivity to mechanical strain, as well as morphological and hormonal factors influencing trabecular adaptation under chronic compartmental loading [11, 14]. Wada et al. assessed proximal tibia MLR using DXA and standing LLXR and found MTP BMD significantly higher than LTP in mild and severe osteoarthritis (OA) [19]. PTE‐MLR was significantly higher in severe OA than in mild OA. In females, PTE‐MLR was significantly higher than in males, corresponding with our findings of significantly higher PTE‐MLR in females. Significant correlations were found between HKAA and PTE‐MLR (*r* = 0.53, *p* < 0.001) [19]. These DXA findings are consistent with the present CT‐HU results.

Importantly, the association between coronal alignment and medial‐to‐lateral bone density ratios persisted after adjustment for age, sex and BMI, supporting alignment as an independent determinant of regional trabecular adaptation rather than a surrogate of demographic or anthropometric factors. Notably, female sex remained independently associated with higher proximal tibial and combined MLR, supporting the sex‐specific differences observed in stratified analyses. These findings suggest that regional trabecular bone adaptation to coronal loading may differ between sexes, beyond differences attributable to alignment severity alone.

Overall, DFE was found to have 28.4% higher BMD than PTE, with only two cases where the opposite was observed. A prior study by Tie et al. measured BMD in tunnel regions for anterior cruciate ligament reconstruction to assess whether femoral tunnel BMD was higher than tibial tunnel BMD [16]. The authors found femoral tunnel BMD higher than tibial tunnel BMD. These findings correspond with those in the present study, which found higher DFE BMD versus PTE BMD. Lower PTE BMD may contribute to the higher reported fracture prevalence of the tibial plateau. A Danish national registry study included 60,823 patients with knee fractures and reported 51% incidence of proximal tibia fractures and 18% incidence of distal femur fractures [18].

In the distal femur, higher mean BMD was observed in the LFC compared with MFC, whereas the opposite pattern was observed in the proximal tibia, with higher mean BMD in the MTP compared with LTP. These compartmental patterns are consistent with prior research. Using DXA, Yoon et al. similarly reported higher BMD in the LFC relative to the MFC and higher density in the medial versus LTP [21]. Prior work using regional knee CT HU as a surrogate for BMD demonstrated the same directional femoral and tibial asymmetries [8]. Together, these findings support a reproducible pattern of compartment‐specific bone adaptation across imaging modalities.

The present findings extend prior results from Rougerou et al., which assessed the relationship between knee alignment and femoral condyle BMD using HKAA and MLR based on CT‐HU measurements [12]. Similar to findings by Rougerou et al., the present study found DFE‐MLR significantly lower in valgus cases versus normal and lowest in the major valgus group (*p* < 0.001). The present study also expands on the work of Rougerou et al., using CT HU, assessing the association between HKAA and PTE‐MLR and measuring HU for entire trabecular areas for both femoral condyle and tibial plateau regions. These results also align with findings by Yoon et al., who studied 122 patients with knee OA undergoing TKA using distal femoral and proximal tibial compartment‐specific DXA and found increasing varus alignment associated with higher medial and lower lateral compartment BMD [21]. Lo et al., in a study of 436 participants using tibial periarticular tibial DXA, also found varus associated with higher tibial‐MLR [10]. DXA‐based MLR assessment is limited by its projectional nature and inability to isolate trabecular bone, limitations addressed by CT.

These regional MLR findings have important implications for TKA and UKA pre‐operative planning. Andersen et al. found that using radiostereometric analysis and DXA for proximal tibia regions, low preoperative BMD was associated with tibia component migration in uncemented TKA [1]. Choice of cemented vs cementless prostheses may be impacted by improved understanding of BMD patterns. Improved preoperative diagnostics for alignment‐associated reductions in MLRs could help guide surgical planning. Early postoperative periprosthetic insufficiency fractures have been reported by Carli et al. in two female siblings with preoperative varus knees who underwent mechanically aligned TKA [3]. Our findings suggest that regional BMD in females may be more susceptible to coronal alignment variations. As Carli et al. noted, knowledge of lower distal femur trabecular density may alter surgical decision‐making, with a preference towards stemmed femoral components [3].

This study has several limitations. First, 99.4% of the study population underwent imaging in preparation for robotic‐assisted hip or knee arthroplasty procedures (knee arthroplasty 70.5%, hip arthroplasty 28.8%), which may limit generalizability. Additionally, the majority of CT scans were obtained in patients with OA. Subchondral sclerosis, which is common in OA [9], can affect BMD measurements [19]. To mitigate this, regions of interest excluded visible sclerosis and averaged HU values across 15 mm of trabecular bone to provide broad epiphyseal sampling. Availability of both CT and standing AP LLXR within a 1‐year period is uncommon outside of hip and knee arthroplasty populations; therefore, these findings may help inform and power future prospective studies, including those involving patients without OA. Second, because this population presented in the setting of OA, it is unknown how altered weight‐bearing secondary to pain may have affected BMD, as gait parameters were unavailable for sub‐analysis. Only two cases of major valgus were identified in the study sample, which limits interpretation for this alignment group. However, similar MLR values were observed in both cases, providing some confidence in these findings. Additional research is warranted for this alignment phenotype. Lastly, despite a relatively large sample of 312 patients, study diversity may have been limited by the surrounding demographics of the study institution. Future research should focus on expanding diversity to allow subgroup analyses.

This study has multiple strengths. To our knowledge, this is the first study to assess medial‐to‐lateral bone density ratios (MLR) using CT HU across the entire distal femoral and proximal tibial epiphyses. The study included a relatively large sequential sample of all eligible patients. Additionally, the cohort consisted of a consecutive institutional sample that was nearly evenly split between sexes (158 females, 154 males). This is notable because bone density research among male populations remains limited [4]. A further strength of this study is the availability of both CT and standing AP long‐leg radiographs within a 1‐year period, a combination that is uncommon outside hip and knee arthroplasty populations. This dataset enabled evaluation of the relationship between coronal alignment and regional trabecular bone density and may help inform and power future prospective studies, including those involving patients without OA.

Coronal knee alignment demonstrated a strong association with compartmental epiphyseal BMD asymmetry on CT. Increasing varus alignment was associated with higher medial trabecular bone density at both the distal femur and proximal tibia, whereas valgus alignment was associated with lower MLRs. The association between HKAA and combined MLR was more pronounced in females, with greater between‐group differences observed compared with males, suggesting sex‐related differences in alignment‐associated trabecular adaptation. Overall, BMD was higher in the DFE than in the PTE. These findings characterize alignment‐dependent regional trabecular bone density patterns of the knee and provide quantitative imaging context relevant to musculoskeletal assessment and arthroplasty planning.

## AUTHOR CONTRIBUTIONS

**Craig E. Klinger**: Conceptualization; methodology; validation; formal analysis; investigation; data curation; writing—original draft preparation; writing—review and editing; visualization; project administration. **Robert E. Bilodeau**: Methodology; resources; writing—original draft preparation; writing—review and editing; supervision. **Maximilian M. Mueller**: Methodology; writing—original draft preparation; writing—review and editing; supervision. **Peter K. Sculco**: Validation; writing—original draft preparation; writing—review and editing; supervision. **Scott A. Rodeo**: Methodology; writing—original draft preparation; writing—review and editing; visualization; supervision. **Elizabeth B. Gausden**: Methodology; writing—original draft preparation; writing—review and editing; visualization; supervision. **Hollis G. Potter**: Methodology; writing—original draft preparation; writing—review and editing; visualization; supervision. **Emily M. Stein**: Methodology; writing—original draft preparation; writing—review and editing; visualization; supervision. **William M. Ricci**: Methodology; resources; writing—original draft preparation; writing—review and editing; visualization; supervision. **Kathryn A. Barth**: Methodology; resources; writing—original draft preparation; writing—review and editing; visualization; supervision. **Derek G. Hansen**: Methodology; resources; writing—original draft preparation; writing—review and editing; visualization; supervision.

## CONFLICT OF INTEREST STATEMENT

Peter K. Sculco consults for BICMD, Inc., Enovis and Zimmer Biomet; receives royalties from Enovis and Zimmer Biomet; research support from Zimmer Biomet; serves on the advisory board of Osgenic; and holds ownership interests in BetterPT, HS2, LLC, HSS ASC Development Network, LLC, Intellijoint Surgical, Inc., Joint Effort Administrative Services Organization, LLC, Parvizi Surgical Innovation, LLC and Osgenic. Scott A. Rodeo consults for Teladoc and holds ownership interests in HS2, LLC, HSS ASC Development Network, LLC, Joint Effort Administrative Services and Ortho RTI. Elizabeth B. Gausden consults for BICMD, Inc. and Stryker Corporation and holds an ownership interest in Joint Effort Administrative Services Organization, LLC. Hollis G. Potter holds advisory or consulting roles with the Atria Institute, Cytex Therapeutics, Inc., Major League Baseball and the National Hockey League; editorial or board roles with the Academy for Radiology & Biomedical Imaging Research, the American Orthopaedic Society for Sports Medicine and *Clinical Orthopaedics and Related Research*; an ownership interest in Imagen Technologies; and research support from the National Institutes of Health, General Electric Healthcare and Siemens Medical Solutions USA, Inc. Emily M. Stein receives research support from Novartis and Radius. William M. Ricci consults for Smith & Nephew; receives royalties from Smith & Nephew, OsteoCentric and Wolters Kluwer; research support from AONA, COTA, the Foundation for Orthopedic Trauma, Metrc and Smith & Nephew; serves on the editorial board of Wolters Kluwer and the board of the Orthopaedic Trauma Association; and holds ownership interests in Cable Fix LLC, CrookedFoot Medical LLC, HS2, LLC, Joint Effort Administrative Services Organization, LLC, McGinley Orthopaedic Innovations, LLC and Primo MC LLC. Derek G. Hansen holds an ownership interest in Joint Effort Administrative Services Organization. The remaining authors declare no conflict of interest.

## ETHICS STATEMENT

This retrospective study was approved by the Hospital for Special Surgery Institutional Review Board (IRB 2023‐1990). This study was approved by our Institutional Review Board, and informed consent was waived due to the retrospective design of this study.

## Supporting information

STROBE_checklist_v4.

Supplemental Material.

Supplemental Tables.

## Data Availability

The data that support the findings of this study are available from the corresponding author upon reasonable request.
