# Supplementary material for: Coronal knee alignment is directly related to knee medial‐to‐lateral bone density ratio
Source: J Exp Orthop. 2026 Apr 14;13(2):e70719. doi: 10.1002/jeo2.70719 (PMC13078134; doi:10.1002/jeo2.70719)
Supplement: Supplementary file 2 — Supplemental Material. [file JEO2-13-e70719-s001.docx]

# The following appendix provides a detailed description of the CT-based Hounsfield Unit measurement protocol to support reproducibility and inter-study comparability.

# APPENDIX

## Hounsfield Unit Measurement Protocol

Region of interest (ROI) Hounsfield Unit (HU) measurements were performed using multiplanar reconstruction (MPR) mode along the axial image plane and included only trabecular bone while excluding cortical bone within the six study ROI areas (Fig. 1). All measurements were performed using Sectra IDS7 PACS (Sectra, Sweden). Measurements were performed using the slab feature within MPR mode, at a slab thickness set at 5mm and step of 5mm using mean HU values. If bone cysts were present within the epiphyseal regions of the distal femur and proximal tibia, they were excluded from the ROI definition. ROIs were defined outside of visible sclerotic bone margins to minimize potential impact of sclerotic bone density on HU measurements. HU values were recorded using a Microsoft Excel datasheet (version 16.100.1; Microsoft Corporation, Redmond, WA), and data recorded included the mean HU value, standard deviation, area (mm^2^), and perimeter (mm). Three HU consecutive measurements were recorded from axial imaging within each of the six study ROI areas (each including 15mm epiphyseal trabecular bone regions). For each ROI, a mean HU value was calculated automatically within the spreadsheet for each of the three consecutive measurements within each ROI area.

1. **Distal femur epiphysis:** Using MPR, the coronal plane was adjusted to align with the distal margin of the medial and lateral femoral condyles, and the sagittal plane to align with the axis of the distal femur including the metaphyseal and epiphyseal aspects. HU measurements began at the postero-superior apex of the femoral intercondylar notch (Fig. 1) and progressed distally on three consecutive images, covering the majority of the designated region while excluding the subchondral plate and any visible sclerotic bone margins. HU measurements of the medial and lateral femoral condyle (detailed below) began at the same axial slice and plane orientation as the distal femur epiphysis HU measurements, using the corresponding axial slices.
2. **Medial and lateral femoral condyles:** The distal femur epiphysis was subdivided into medial femoral condyle and lateral femoral condyle (Fig. 1). This was accomplished using the slice corresponding with the most proximal slice measured for the distal femur epiphysis. A 90º angle was drawn with the angle feature in PACS, with a line adjacent to the posterior margin of both femoral condyles. Two lines were then drawn with orientation matching the 90º perpendicular line and then were placed along the medial and lateral margins of the femoral condyle. The width was measured of the medial to lateral distance the line tool in PACS with the line orientation matching the posterior femoral condyle line. A second line was drawn along the same orientation exactly half of the length of the prior line to establish the central point between medial and lateral condyles and the 90º perpendicular line was moved for reference to serve as a dividing line for medial and lateral femoral condyle measurements. HU measurements were taken on three consecutive slices, distally.
3. **Proximal tibia epiphysis:** Using MPR, the coronal plane was adjusted to align with the distal margin of the medial and lateral tibial plateau subchondral plate, and the sagittal plane to align with the tibial axis. For the proximal tibia epiphysis, HU measurement started below the level of the subchondral plate and below visible sclerotic bone margins (Fig. 1) and progressed distally on three consecutive slices. HU measurements of the medial and lateral tibial plateaus (detailed below) began at the same axial slice and plane orientation as the proximal tibia epiphysis HU measurements, using the corresponding axial slices.
4. **Medial and lateral tibial plateaus:** The proximal tibia epiphysis was subdivided into medial tibial plateau and lateral tibial plateau (Fig. 1). This was accomplished using the slice corresponding with the most proximal slice measured for the proximal tibia epiphysis. An 90º angle was drawn using the angle feature in PACS, with a line adjacent to the posterior margin of proximal tibia. Two lines were then drawn with orientation matching the 90º perpendicular line and then were placed along the medial and lateral margins of the proximal tibia. The width was measured of the medial to lateral distance the line tool in PACS with the line orientation matching the posterior proximal tibia line. A second line was drawn along the same orientation exactly half of the length of the prior line to establish the central point between medial and lateral tibial plateaus and the 90º perpendicular line was moved for reference to serve as a dividing line for medial and lateral tibial plateau measurements. HU measurements were taken on three consecutive slices, distally.
