# Supplementary material for: Coronal knee alignment is directly related to knee medial‐to‐lateral bone density ratio
Source: J Exp Orthop. 2026 Apr 14;13(2):e70719. doi: 10.1002/jeo2.70719 (PMC13078134; doi:10.1002/jeo2.70719)
Supplement: Supplementary file 3 — Supplemental Tables. [file JEO2-13-e70719-s003.docx]

| **Supplemental Table 1.** Medial-to-lateral ratio pairwise comparisons by alignment group. | | | | | | |
| --- | --- | --- | --- | --- | --- | --- |
|  |  |  |  |  |  |  |
|  | **DFE-MLR** | | **PTE-MLR** | | **C-MLR** | |
| **Sample 1-Sample 2** | **p-value** | **Adj.  p-value** | **p-value** | **Adj.  p-value** | **p-value** | **Adj. p-value** |
| Major Valgus-Valgus | 0.384 | >0.999 | 0.348 | >0.999 | 0.485 | >0.999 |
| Major Valgus-Neutral | 0.080 | 0.803 | 0.039 | 0.386 | 0.070 | 0.700 |
| Major Valgus-Varus | 0.009 | 0.086 | 0.015 | 0.146 | 0.009 | 0.090 |
| Major Valgus-Major Varus | 0.006 | 0.057 | 0.004 | **0.042** | 0.003 | **0.029** |
| Valgus-Neutral | 0.004 | **0.043** | <.001 | **<0.001** | <.001 | **0.003** |
| Valgus-Varus | <.001 | **<0.001** | <.001 | **<0.001** | <.001 | **<0.001** |
| Valgus-Major Varus | <.001 | **<0.001** | <.001 | **<0.001** | <.001 | **<0.001** |
| Neutral-Varus | <.001 | **0.001** | 0.104 | >0.999 | <.001 | **0.003** |
| Neutral-Major Varus | <.001 | **0.001** | 0.003 | **0.027** | <.001 | **<0.001** |
| Varus-Major Varus | 0.439 | >0.999 | 0.040 | 0.397 | 0.067 | 0.670 |
| *Independent-Samples Kruskal-Wallis Test.* | | | | | | |
| *DFE=distal femur epiphysis, PTE=proximal tibia epiphysis, MLR=medial-to-lateral ratio,* | | | | | | |
| *C-MLR=Combined MLR, Adj. p-value=adjusted p-value; by Bonferroni correction.* | | | | | | |
|  |  |  |  |  |  |  |

| **Supplemental Table 2.** MLR BMD measurements by knee alignment group stratified by sex. | | | | |
| --- | --- | --- | --- | --- |
|  |  |  |  |  |
| **HKAA Group** | | | **N (%)** | **Median (IQR)** |
| **Females: Distal Femur Epiphysis MLR (N=158)** | | | |  |
|  | **Overall** | | **158 (100)** | **0.83 (0.73-0.93)** |
|  |  | Major Varus | 19 (12.0) | 0.93 (0.83-1.01) |
|  |  | Varus | 69 (43.7) | 0.90 (0.81-0.98) |
|  |  | Neutral | 43 (27.2) | 0.78 (0.70-0.87) |
|  |  | Valgus | 26 (16.5) | 0.71 (0.58-0.77) |
|  |  | Major Valgus | 1 (0.6) | 0.60 (0.60-0.60) |
| **Males: Distal Femur Epiphysis MLR (N=154)** | | | |  |
|  | **Overall** | | **154 (100)** | **0.84 (0.77-0.92)** |
|  |  | Major Varus | 34 (22.1) | 0.86 (0.79-0.92) |
|  |  | Varus | 98 (63.6) | 0.84 (0.78-0.94) |
|  |  | Neutral | 14 (9.1) | 0.83 (0.72-0.87) |
|  |  | Valgus | 7 (4.5) | 0.73 (0.67-0.83) |
|  |  | Major Valgus | 1 (0.6) | 0.49 (0.49-0.49) |
| **Females: Proximal Tibia Epiphysis MLR (N=158)** | | | |  |
|  | **Overall** | | **158 (100)** | **1.39 (1.21-1.71)** |
|  |  | Major Varus | 19 (12.0) | 1.63 (1.42-2.13) |
|  |  | Varus | 69 (43.7) | 1.52 (1.33-1.81) |
|  |  | Neutral | 43 (27.2) | 1.29 (1.15-1.52) |
|  |  | Valgus | 26 (16.5) | 1.08 (0.91-1.23) |
|  |  | Major Valgus | 1 (0.6) | 0.96 (0.96-0.96) |
| **Males: Proximal Tibia Epiphysis MLR (N=154)** | | | |  |
|  | **Overall** | | **154 (100)** | **1.22 (1.10-1.40)** |
|  |  | Major Varus | 34 (22.1) | 1.37 (1.18-1.73) |
|  |  | Varus | 98 (63.6) | 1.22 (1.09-1.37) |
|  |  | Neutral | 14 (9.1) | 1.19 (1.11-1.26) |
|  |  | Valgus | 7 (4.5) | 1.02 (0.94-1.16) |
|  |  | Major Valgus | 1 (0.6) | 0.64 (0.64-0.64) |
| **Females: Combined-MLR (N=158)** | | |  |  |
|  | **Overall** | | **158 (100)** | **1.03 (0.92-1.16)** |
|  |  | Major Varus | 19 (12.0) | 1.20 (1.04-1.33) |
|  |  | Varus | 69 (43.7) | 1.09 (1.02-1.20) |
|  |  | Neutral | 43 (27.2) | 0.97 (0.89-1.05) |
|  |  | Valgus | 26 (16.5) | 0.84 (0.75-0.93) |
|  |  | Major Valgus | 1 (0.6) | 0.71 (0.71-0.71) |
| **Males: Combined MLR (N=154)** | | |  |  |
|  | **Overall** | | **154 (100)** | **0.99 (0.92-1.06)** |
|  |  | Major Varus | 34 (22.1) | 1.06 (0.96-1.15) |
|  |  | Varus | 98 (63.6) | 0.99 (0.91-1.06) |
|  |  | Neutral | 14 (9.1) | 0.95 (0.94-0.99) |
|  |  | Valgus | 7 (4.5) | 0.87 (0.77-0.93) |
|  |  | Major Valgus | 1 (0.6) | 0.55 (0.55-0.55) |
| *MLR=medial-to-lateral ratio, BMD=bone mineral density,* | | | | |
| *HKAA=hip-knee-ankle angle, IQR=interquartile range (IQR, 25% to 75%).* | | | | |

| **Supplemental Table 3.** Correlations between variables stratified by sex. | | | | |
| --- | --- | --- | --- | --- |
|  |  |  |  |  |
|  | **Females** | | **Males** | |
| **Variables** | **Correlation Coefficient*** | **p-value** | **Correlation Coefficient*** | **p-value** |
| HKAA and DFE-MLR | -0.570 | **<.001** | -0.307 | **<.001** |
| HKAA and PTE-MLR | -0.410 | **<.001** | -0.481 | **<.001** |
| DFE-MLR and PTE-MLR | 0.359 | **<.001** | 0.296 | **<.001** |
| Combined-MLR and HKAA | -0.631 | **<.001** | -0.478 | **<.001** |
| Bodyweight and HKAA | -0.283 | **<.001** | -0.115 | 0.157 |
| Height and HKAA | 0.104 | **0.194** | -0.035 | **0.670** |
| BMI and HKAA | -0.347 | **<.001** | -0.097 | 0.231 |
| Age and DFE-MLR | -0.125 | **0.117** | -0.357 | **<.001** |
| Age and PTE-MLR | 0.104 | **0.192** | 0.082 | **0.309** |
| Age and Combined-MLR | 0.022 | 0.781 | -0.179 | **0.027** |
| Age and MFC HU | -0.222 | **0.005** | -0.261 | **0.001** |
| Age and LFC HU | -0.177 | **0.027** | -0.116 | 0.153 |
| Age and MTP HU | -0.166 | **0.037** | -0.236 | **0.003** |
| Age and LTP HU | -0.019 | 0.814 | 0.044 | 0.585 |
| Bodyweight and MFC HU | 0.142 | **0.075** | 0.183 | **0.023** |
| Bodyweight and LFC HU | 0.111 | 0.166 | 0.157 | 0.051 |
| Bodyweight and MTP HU | 0.186 | **0.020** | 0.227 | **0.005** |
| Bodyweight and LTP HU | 0.114 | 0.154 | 0.159 | **0.049** |
| Height and MFC HU | -0.012 | **0.881** | -0.007 | **0.936** |
| Height and LFC HU | -0.010 | **0.901** | 0.019 | **0.816** |
| Height and MTP HU | -0.033 | **0.676** | -0.003 | **0.966** |
| Height and LTP HU | -0.019 | **0.814** | 0.044 | **0.585** |
| BMI and MFC HU | 0.161 | **0.043** | 0.197 | **0.014** |
| BMI and LFC HU | 0.128 | **0.110** | 0.147 | **0.068** |
| BMI and MTP HU | -0.166 | **0.037** | -0.236 | **0.003** |
| BMI and LTP HU | 0.140 | **0.080** | 0.137 | **0.090** |
| **Pearson correlation coefficient, HKAA=hip-knee-ankle angle,* | | | | |
| *DFE=distal femur epiphysis, PTE=proximal tibia epiphysis,* | | | | |
| *MLR=medial-to-lateral ratio, Combined-MLR=MFC+MTP to LFC+LTP ratio,* | | | | |
| *MFC=medial femoral condyle, LFC=lateral femoral condyle,* | | | | |
| *MTP=medial tibial plateau, LTP=lateral tibial plateau, HU=Hounsfield Units,* | | | | |
| *BMI=body mass index.* | | | | |

| **Supplemental Table 4.** Medial-to-lateral pairwise comparisons by alignment group stratified by sex. | | | | | | | | | | | | |
| --- | --- | --- | --- | --- | --- | --- | --- | --- | --- | --- | --- | --- |
|  |  |  |  |  |  |  |  |  |  |  |  |  |
|  | **Females** | | | | | | **Males** | | | | | |
|  | **DFE-MLR** | | **PTE-MLR** | | **C-MLR** | | **DFE-MLR** | | **PTE-MLR** | | **C-MLR** | |
| **Sample 1-Sample 2** | **p-value** | **Adj. p-value** | **p-value** | **Adj. p-value** | **p-value** | **Adj. p-value** | **p-value** | **Adj. p-value** | **p-value** | **Adj. p-value** | **p- value** | **Adj. p-value** |
| Major Valgus-Valgus | 0.615 | >0.999 | 0.626 | >0.999 | 0.692 | >0.999 | 0.366 | >0.999 | 0.493 | >0.999 | 0.516 | >0.999 |
| Major Valgus-Neutral | 0.254 | >0.999 | 0.203 | >0.999 | 0.240 | >0.999 | 0.172 | >0.999 | 0.168 | >0.999 | 0.219 | >0.999 |
| Major Valgus-Varus | 0.054 | 0.538 | 0.053 | 0.528 | 0.041 | 0.412 | 0.079 | 0.788 | 0.094 | 0.938 | 0.088 | 0.877 |
| Major Valgus-Major Varus | 0.042 | 0.421 | 0.037 | 0.371 | 0.026 | 0.261 | 0.062 | 0.617 | 0.032 | 0.320 | 0.034 | 0.337 |
| Valgus-Neutral | 0.010 | 0.099 | 0.001 | **0.015** | 0.002 | **0.016** | 0.336 | >0.999 | 0.134 | >0.999 | 0.213 | >0.999 |
| Valgus-Varus | <.001 | **<0.001** | <.001 | **<0.001** | <.001 | **<0.001** | 0.041 | 0.410 | 0.015 | 0.151 | 0.009 | 0.090 |
| Valgus-Major Varus | <.001 | **<0.001** | <.001 | **<0.001** | <.001 | **<0.001** | 0.025 | 0.253 | <.001 | **0.005** | <0.001 | **0.004** |
| Neutral-Varus | <.001 | **<0.001** | <.001 | **0.006** | <.001 | **<0.001** | 0.215 | >0.999 | 0.368 | >0.999 | 0.119 | >0.999 |
| Neutral-Major Varus | <.001 | **0.007** | 0.002 | **0.020** | <.001 | **0.001** | 0.128 | >0.999 | 0.018 | 0.184 | 0.005 | 0.054 |
| Varus-Major Varus | 0.580 | >0.999 | 0.468 | >0.999 | 0.384 | >0.999 | 0.516 | >0.999 | 0.014 | 0.136 | 0.028 | 0.278 |
| *Independent-Samples Kruskal-Wallis Test, DFE=distal femur epiphysis, PTE=proximal tibia epiphysis, MLR=medial-to-lateral ratio,* | | | | | | | | | | | | |
| *C-MLR=combined-MLR, Adj. p-value=adjusted p-value; by Bonferroni correction.* | | | | | | | | | | |  |  |
